# Usability Acceptability and Satisfaction of a New Mobile Intervention among Nepalese Women with Gestational Diabetes: A Cross-sectional Study

**DOI:** 10.31729/jnma.v63i290.9201

**Published:** 2025-09-01

**Authors:** Kalpana Chaudhary, Aarthi Shanmugavel, Bipsana Shrestha, Jyoti Nepal, Bhawana Shrestha, Pratiksha Paudel, Kusum Shrestha, Abha Shrestha, Shanti Subedi, Prabin Shakya, Dipesh Khadka, Jean-Francois Daneault, Archana Shrestha, Shristi Rawal

**Affiliations:** 1 Department of Public Health, Kathmandu University School of Medical Sciences, Dhulikhel, Nepal; 2 School of Health Professions, Rutgers University, New York, New Jersey, United States; 3 Institute of Medicine, Tribhuvan University, Maharajgunj, Nepal; 4 Department of Research and Development, Dhulikhel Hospital, Kathmandu University Hospital, Dhulikhel, Nepal; 5 Department of Obstetrics and Gynaecology, Dhulikhel Hospital, Kathmandu University Hospital, Dhulikhel, Nepal; 6 Department of Obstetrics and Gynaecology, Nobel Medical College & Teaching Hospital, Biratnagar, Nepal; 7 Biomedical Knowledge Engineering Lab, Seoul National University, Seoul, Korea

**Keywords:** *gestational diabetes*, *mHealth app*, *diabetes intervention*

## Abstract

**Introduction:**

Gestational diabetes mellitus is among the most common pregnancy complications with various adverse maternal and fetal outcomes. Mobile health technology offers new opportunities to enhance its care and support self-management. The aim of study was to identify usability, acceptability and satisfaction of ‘Garbhakalin Diabetes athawa Madhumeha-Dhulikhel Hospital’ application to support the treatment and self-management of gestational diabetes mellitus among Nepalese women.

**Methods:**

A cross-sectional study among 46 women of an intervention arm of a parent randomized controlled trial was conducted. The ethical/institutional review boards of Rutgers University (Pro2019001883) and the Nepal Health Research Council approved the study (Ref number: 735/2019). Perceived usability and acceptability of the application was assessed using the System Usability Scale and mobile Health Application Usability Questionnaire. To assess satisfaction with gestational diabetes mellitus care, the Oxford Maternity Diabetes Treatment Satisfaction Questionnaire was used. Frequencies and percentages are reported for categorical variables, and means and standard deviation for continuous variables.

**Results:**

The average time spent on the application was 37 minutes per day, with 12 minutes of engagement per session. The mean score on System Usability Scale was 72.12±4.78; 44 (95.56%) participants liked using the application, 45 (97.76%) found it easy, and 40 (86.91%) praised its functional integration. The mean score on the mobile Health Application Usability Questionnaire and Oxford Maternity Diabetes Treatment Satisfaction Questionnaire were 118.32±8.84 and 15.82±4.09 respectively.

**Conclusions:**

The mobile application demonstrated strong usability and was well-accepted by Nepalese women with gestational diabetes mellitus, suggesting it is a promising tool for self-management support.

## INTRODUCTION

Gestational diabetes mellitus (GDM), one of the most common pregnancy complications^1,2^ with adverse outcomes like preeclampsia, birth injuries,^3-5^ are preventable through timely management of GDM.^6-8^ Most women maintain glycemic control through dietary changes and regular exercise with or without medication.^6-9^ However, women fail to find appropriate support and receive inconsistent information^10^ in resource-constrained settings like Nepal.^1^

Mobile health (mHealth) technology can support GDM self-management in Nepalese women^12^ with mobile service penetration exceeding 100%.^13^ At Dhulikhel Hospital, the ‘Garbhakalin Diabetes athawa Madhumeha-Dhulikhel Hospital’ (GDM-DH); (first application to manage GDM in a low-resource setting) was developed and optimized using "think-aloud" protocol.^14^ Grounded in social cognitive theory,^15^ it aids GDM self-management by providing educational resources and allowing users to monitor blood glucose, blood pressure, carbohydrate intake, physical activity, and gestational weight gain. Its user-centered approach effectively addresses the needs of users.^16-18^ Our study assessed its usability, acceptability and satisfaction among Nepalese women.

## METHODS

A cross-sectional study among 46 women of an intervention arm of a parent randomized controlled trial was conducted. A total of 54 women were enrolled in the intervention arm however, five of them dropped out of the study (1 mis-carriage and 4 discontinued using the mobile app) and three more were excluded after assessing for eligibility. The parent study was a pilot feasibility RCT (Trial Registration number: NCT04198857) in which women with GDM were assigned to one of two treatment conditions: either (A) GDM-DH app plus standard care or (B) standard care alone, starting from diagnosis in the 3^rd^ trimester (28-30 gestational weeks) until delivery. The development and features of the GDM-DH application have been described in detail previously.^19^ All pregnant women receiving antenatal care at Obstetric Outpatient Department (OPD) ofDhulikhel Hospital, Kathmandu University Hospital underwent routine screening for GDM at 24-28 weeks of gestation. Patients received a diagnosis of GDM based on the Carpenter Coustan criteria. One hundred twelve eligible pregnant women with GDM were recruited in the RCT from October 2021 to June 2024. Of these participants, we assessed the feasibility outcomes among 46 women with GDM in the intervention group. The ethical/institutional review boards of Rutgers University (Pro2019001883) and the Nepal Health Research Council approved the study (Ref number: 735/2019). Written informed consent was obtained from the participants. Women aged 18 years and above, newly diagnosed with GDM, less than 30 weeks pregnant, owned an Android smartphone, had internet connectivity at home, and could understand and read Nepali were included in the study. Patients with learning difficulties or vision/hearing impairments and patients previously diagnosed with diabetes and multifetal pregnancy were excluded. App usage was monitored among participants through Google Analytics via Firebase. They included average engagement time, average engagement time per session, and the number of times the app and each component were opened and viewed (total across participants). Participants’ devices automatically reported the data to Google Analytics. Perceived usability and acceptability of the app was assessed using the System Usability Scale (SUS), a 10-item 5-point Likert scale questionnaire focused on usability and human-computer interaction,^20^ as well as 21-item mHealth App Usability Questionnaire (MAUQ). MAUQ is a comprehensive tool for evaluating the usability of mHealth applications, focusing on ease of use, satisfaction, usefulness, and information quality. Each item is rated on a 7-point Likert scale ranging from "Strongly Disagree" (1) to "Strongly Agree" (7). The subscales included Ease of Use and Satisfaction (8 items, MAUQ_E), System Information Arrangement (6 items, MAUQ_S), and Usefulness (7 items, MAUQ_U). Their respective Cronbach's alpha values were 0.895, 0.829, and 0.900, demonstrating internal solid consistency. The scores for each subscale are averaged to provide an overall usability score. Higher scores indicate better usability, meaning the app is perceived as easy to use, effective, and satisfactory.^21^ To evaluate satisfaction with GDM care, the Oxford Maternity Diabetes Treatment Satisfaction Questionnaire (OMDTSQ)^22^ was used, a 9-item validated tool for assessing general satisfaction and the acceptability of technology in diabetes care. We asked women to rate their agreement with statements on a 7-point Likert scale, ranging from +3 (strongly agree) to -3 (strongly disagree). In the main study, the SUS, MAUQ and OMDTSQ responses showed good /acceptable internal consistency, with a Cronbach’s alpha of 0.60, 0.79, and 0.89, respectively. Data were collected in REDCap and exported to STATA version 18. Quantitative data are presented in frequencies and percentages for categorical variables, and in means and standard deviation for continuous variables.

## RESULTS

The average age of the participants was 29.91±4.13 years, with 48 (88.84%) participants identifying as Hindu and 25 (46.34%) belonging to the Brahmin/Chhetri and 21 (38.91%) belonging to Newar ethnic groups. Half of the women were homemakers. On average, participants had a family size of 4.92±2.33 members. There were 39 (72.23%) multigravida participants and 13 (35.38%) with previous caesarean delivery. At the time of the interview, the average gestational age was 27±2.33 weeks. The participants had, on average, 6.21±2.54 antenatal care visits.

**Table 1:** Baseline characteristics of the initial study participants in the intervention group (n=54).

Characteristics	n(%)
**Education**	
Primary	2(3.67)
Secondary level	28(51.85)
Higher secondary and above	24(44.44)
**Ethnicity**	
Brahmin/Chhetri	25(46.34)
Newar	21(38.91)
Janajati	3(5.56)
Others	5(9.19)
**Religion**	
Hindu	48(88.84)
Buddhist	5(9.24)
Christian	1(1.92)
**Occupation**	
Service	16(29.63)
Business	11(20.37)
Homemaker	27(50.00)
**Gravida**	
Primigravida	15(27.77)
Multigravida	39(72.23)
Previous Caesarean delivery	13(35.38)
Intended Pregnancy	45(83.33)

Users spent an average of 37 minutes daily on the app, with each session lasting 12 minutes of active engagement. There were 44 (95.56%) participants reporting that they would like to use the GDM-DH app frequently and 43 (97.76%) found it easy to use. The app’s various functions were noted to be well integrated by 40 (86.91%) participants and 43 (93.42%) felt confident using it. Only 2 (4.31%) participants considered the app unnecessarily complex. However, 11 (23.92%) participants indicated needing technical assistance to use the app, and 13 (28.23%) mentioned they would need to learn a lot before using it. The mean SUS score was 72.12±4.78 (total possible score of 100).

**Table 2:** System Usability Scale (SUS) of participants (n=46).

System Usability Scale	Mean±SD	Positive Response, n(%)
I think that I would like to use this app frequently	3.11±0.42	44(95.56)
I found the app unnecessarily complex	3.12±0.42	2(4.31)
I thought the app was easy to use	3.00±0.22	45(97.76)
I think that I would need the support of a technical person to be able to use this app	2.74±0.63	11(23.92)
I found the various functions in this app were well integrated	2.93±0.51	40(86.91)
I thought there was too much inconsistency in this app	3.00±0.22	-
I would imagine most people would learn to use this app very quickly	2.78±0.54	36(78.21)
I found the app very cumbersome to use	2.72±0.63	5(10.82)
I felt very confident using the app	2.91±0.42	43(93.42)
I needed to learn a lot of things before I could get going with this app	2.33±0.84	13(28.23)
Total mean score	72.12±4.78	

Scores for individual questions range from 1=Strongly disagree to 5=Strongly agree. To compute the total SUS score for each respondent, we first converted the score for each question to a range from 0 to 4, with scores of 3 and 4 considered positive responses and 4 being the most positive response. To do this, we subtracted one from the user response for all odd-numbered questions, which were positively worded, and for even-numbered items, which were negatively worded, we subtracted the user response from 5. We then added up the converted responses for each user and multiplied that total by 2.5. This scaled the range of possible values from 0 to 100 instead of from 0 to 40, with each question weighing 10 points.

The mean mHealth app usability score was 118.32±8.84. Based on MAUQ, all participants found the GDM-DH app easy to use and well-organized, with 45 (97.82%) expressing a desire to use the app again. Further, 42 (91.34%) participants indicated that the app provided an acceptable way to receive healthcare services, 44 (95.62%) felt that it effectively acknowledged and informed them about the progress of their actions, and 40 (86.94%) were able to recover quickly from mistakes made while using the app. Regarding usefulness, 33 (73.32%) found the app convenient for communicating with their healthcare provider, and 42 (91.32%) reported that it improved their access to healthcare services. Altogether, 32 (68.78%) participants felt that the app offered opportunities for interaction with their healthcare provider, and 28 (62.23%) were confident that their provider would receive any information they sent.

Overall, participants reported satisfaction with diabetes care, the diabetes care team and the technology and system used to manage gestational diabetes. One woman (2.12%) rated their satisfaction with care negatively, including her perceived relationship with the diabetes care team and acceptability and reliability of the technology. Similarly, one woman (2.20%) provided neutral responses in one aspect of the treatment satisfaction characteristics. The mean diabetes treatment satisfaction score was 15.82±4.09.

## DISCUSSION

In this study, the usability of our culturally tailored GDM-DH app was assessed. The app was developed specifically for managing GDM in a low-income country setting. The app’s feasibility was measured through user engagement, system usability, and satisfaction with diabetes treatment. Findings showed that the app was well-received with the participants completing the usability assessment at 37-38 weeks of gestation. The app demonstrated strong usability and acceptability. Women attending Dhulikhel hospital found the app easy to use and well-integrated into their healthcare routines. Many users were confident in using the app and showed a desire to use it frequently which reflected its perceived value and convenience. Additionally, responses to the OMDTSQ revealed high levels of satisfaction with the care received.

The app seemed feasible to use as there was a 90% recruitment rate of eligible participants in the intervention. This is aligned with previous research findings which also reported high participant recruitment.^23^ The high app usage in our study might be due to the user-centred design approach incorporated in developing the GDM-DH app.^16,19^ Collaboration with patients and providers at Dhulikhel Hospital was done to ensure the app met clinical needs and technological sophistication. Further, use of the local language in app may have enhanced engagement among women in the intervention.

**Table 3:** App usability ratings in the intervention group (n=46).

Health app usability questionnaire (MAUQ)		Mean±SD	Positive Response, n(%)
Ease of use and satisfaction	The app was easy to use.	6.00±0.31	45(97.76)
	It was easy for me to learn to use the app.	5.94±0.52	43(97.72)
	I like the interface of the app.	5.94±0.63	44(97.81)
	The information in the app was well organized, so I could easily find the information I needed.	5.92±0.63	46(100.00)
	I feel comfortable using this app in social settings.	5.82±0.53	45(97.76)
	The amount of time involved in using this app has been fitting for me.	5.26±0.82	41(89.12)
	I would use this app again.	6.00±0.63	45(97.76)
	Overall, I am satisfied with this app.	5.93±0.44	45(97.76)
System Information arrangement	Whenever I made a mistake using the app, I could recover easily and quickly.	5.33±1.12	40(86.94)
	This mHealth app provided an acceptable way to receive health care services.	5.56±0.71	42(91.34)
	The app adequately acknowledged and provided information to let me know the progress of my action.	5.71±0.64	44(95.62)
	The navigation was consistent when moving between screens.	5.64±0.92	44(95.56)
	The interface of the app allowed me to use all the functions (such as entering information, responding to reminders, viewing information) offered by the app.	5.72±0.53	45(97.76)
	This app has all the functions and capabilities I expect it to have.	5.44±0.82	38(82.57)
Usefulness	The app would be useful for my health and well-being.	5.92±0.56	43(95.54)
	The app improved my access to healthcare services.	5.62±0.71	42(91.32)
	The app helped me manage my health effectively.	5.94±0.54	46(100.00)
	The app made it convenient for me to communicate with my healthcare provider.	5.00±1.21	33(73.32)
	Using the app, I had many more opportunities to interact with my healthcare provider.	4.83±1.32	31(68.78)
	I felt confident that any information I sent to my app provider would be received and processed.	4.61±1.54	28(62.23)
	I felt comfortable communicating with my healthcare provider using the app.	4.67±1.63	31(68.81)
	Total Mean Score	118.32±8.84	

Scores for individual questions range from 1=Strongly disagree to 7=Strongly. agree. The means and SD for personal statements and the entire questionnaire for the MAUQ were calculated. Scores ranged from 21-147, with higher scores indicating higher app usability. Scores of 5, 6, and 7 were considered positive responses, with 7 being the most positive.

On average, participants spent 37 minutes per day on the app. They reported overall satisfaction with the GDM-DH app, noting that it was easy to use. Around 98% of participants reported that they are willing to use the service again in future pregnancies. Participants said that they found the app helpful in managing their diabetes; most of them indicating that it was convenient as well as reliable. The app helped them in managing their health effectively and making them feel comfortable to communicate with their healthcare provider.

Despite people finding it very easy to use (97.76%) and confident (93.42%) about using it, a substantial proportion also said that they need technical support (23.92%) and that they needed to learn a lot. Similar results were found in mobile health (mHealth) research reporting high usability but requiring technical support^24^ mainly due to difference in digital literacy levels of users.^25^ Incorporating built-in tutorials, individualized guidance like features will help narrow the gap between usability and learnability, which is imperative for digital health tools aiming to manage chronic diseases.^26^

**Table 4:** Oxford Maternity Diabetes Treatment Satisfaction ratings of study participants (n=46).

Satisfaction Questionnaire			disagree/disagree (-3) (-2)	disagree (-1)	(0)	agree (+1)		agree (+3)
General satisfaction with diabetes care	I am satisfied with my current treatment	1.91±0.50	-	-	-	7(15.23)	33(71.71)	6(13.00)
	I am satisfied the treatment I am receiving is the best for me	1.82±0.56	-	-	1(2.20)	11(24.21)	27(60.00)	6(13.31)
	I am satisfied with my understanding of diabetes	1.83±0.62	-	-	-	11(23.91)	29(63.00)	6(13.13)
Perceived relationship with the diabetes care team	I feel my maternity diabetes team knows enough about my current level of diabetes control	1.60±0.91	-	1(2.12)	2(4.31)	14(30.36)	27(58.70)	2(4.40)
I feel I have a good relationship with my maternity diabetes team.	1.62±0.71	1(2.21%)		2(4.31)	12(26.14)	26(56.54)	5(10.89)
	I am satisfied with my maternity diabetes team’s understanding of my diabetes	1.23±0.90	-	-	2(4.42)	18(39.13)	22(47.82)	4(8.72)
Acceptability and reliability of the technology/satisfaction with the GDM health system	I find the equipment I use to check my blood sugars is convenient	1.93±0.51	-	-	1(2.22)	6(13.00)	34(73.89)	5(10.81)
	I feel the equipment I use to check my blood sugars is reliable	1.92±0.47	-	-	-	7(15.19)	34(73.89)	5(10.94)
	My blood sugar monitoring fits in with my lifestyle	1.52±0.81	-	1(2.12)	-	9(19.56)	33(71.67)	3(6.54)
Total Score		15.82±4.09						

Scores for individual questions range from -3=Strongly disagree to +3=Strongly agree. The means and SD for personal statements and the total score were computed, and the questionnaires for the OMDTSQ, with higher scores indicating higher diabetes treatment satisfaction.

In our study, the OMDTSQ responses showed that women were satisfied with their care. These findings are consistent with previous research on women with GDM conducted in the UK.^22^ Both studies utilized a structured OMDTSQ questionnaire to assess women’s satisfaction with diabetes care, with a similar proportion of nulliparous women. In the comparison study, no women gave the most negative scores. However, in our study, few participants responded negatively, particularly about their relationship with the maternity diabetes team, their knowledge of current diabetes control, and how blood sugar monitoring fits into their lifestyle. In low- and middle-income countries (LMICs), maternity diabetes care teams face several challenges like high patient loads and inadequate consultation time, leading to unsatisfactory patient experiences. For instance, doctors in Nepal may see up to 100 patients in a half-day clinic, leaving just 5-10 minutes per case. This leads to restriction in meaningful counseling on lifestyle, diet, and medication, consequently there is poor understanding and mismanagement of the health condition by patients.^27^ These negative satisfaction ratings for diabetes treatment care implies that it is necessary to enhance the relationship among patients, health workers, and technology. In this study, SUS score for the GDM-DH app was 72.12, which falls within the range of good usability according to the previous literature.^28^ Another study on similar digital health interventions for chronic disease management report SUS mean scores between 68-72,^29^ indicating the user satisfaction and perceived ease of use similar to ours.

One of the strengths of our study is being the first RCT study in Nepal to assess the feasibility and acceptability of a mobile app intervention among pregnant women with GDM. In addition, we used validated questionnaires to assess different areas of the feasibility of the app. However, the study has several limitations. First, as a feasibility study, our sample size was limited, so our findings cannot be generalized. More extensive studies involving larger numbers of participants are needed to confirm our results. Second, we included only literate women aged 18 and above with Android phones, which restricts the generalizability of our findings to other populations. Third, while the SUS and MAUQ are validated in various countries, they have yet to be explicitly validated in Nepal. To address this, we translated the SUS and MAUQ questionnaires into Nepali and then translated them back into English. We also conducted cognitive testing with a small sample of target users during the pilot phase to ensure the tool’s appropriateness and ease of interpretation. In the future, we aim to enhance the GDM-DH app by integrating Bluetooth-enabled data entry, multimedia push notifications, and gamification elements, which have been shown to improve user retention and engagement in mobile health (mHealth) interventions. Push notifications facilitate the timely delivery of content, providing essential triggers and reinforcement when needed. Gamification features, such as points, badges, levels, and leader boards, will be incorporated to offer motivation and entertainment, encouraging sustained engagement.^19^

## CONCLUSION

The GDM-DH App is a feasible, user-friendly, and well-accepted tool among Nepalese women with GDM. The clinical effectiveness of this study is currently under evaluation, while implementation research remains crucial for understanding its impact on outcomes, refining the standard of care, and enhancing clinician-patient communication. Closing these gaps through targeted study is essential for advancing healthcare delivery and improving patient well-being.

## Data Availability

The data are available from the corresponding author upon reasonable request.

## Trial Registration number of the parent study

Registered in ClinicalTrials.gov, Trial Registration number-NCT04198857 (First Posted 2019-12-13)
